# A Survey of Knowledge, Clinical Practice, and Barriers Related to Sarcopenia in Korean Physical Therapists: A Cross-Sectional Survey

**DOI:** 10.3390/healthcare14070921

**Published:** 2026-04-01

**Authors:** Jaehyun Lim, Byeonggeun Kim, Ahyoung Choi

**Affiliations:** 1 Department of Physical Therapy, Graduate School, Nambu University, Gwangju 62271, Republic of Korea; jhjhoss@naver.com; 2 Regional Health & Medical Center for Persons with Disabilities, Chonnam National University Hospital, Gwangju 61469, Republic of Korea; qudrms_92@naver.com; 3 Department of Health and Safety Management, Songwon University, Gwangju 61756, Republic of Korea

**Keywords:** Korean physical therapist, sarcopenia, survey, knowledge, clinical practice, barrier

## Abstract

**Background/Objectives**: Rapid population aging has increased the need for effective sarcopenia management. Physical therapists are expected to play a key role in screening and intervention. This study aimed to assess sarcopenia awareness, current clinical practice, and barriers among Korean physical therapists to inform future education and policy improvements. **Methods**: A cross-sectional survey was conducted in Korea from 14 August to 31 December 2025. The questionnaire was developed through a two-step process, including a qualitative survey and cognitive interviews with physical therapists, and consisted of items on demographics, sarcopenia knowledge, screening, treatment, and care environment factors. **Results**: A total of 236 participants were included in the analysis. Although awareness of sarcopenia was high, implementation in clinical practice remained limited. Most participants were familiar with sarcopenia and considered it important in patient management, yet only 16.9% reported screening for sarcopenia and 23.7% reported providing treatment. Among those who performed screening, half were unsure which guideline was being followed. Resistance exercise was the most commonly used intervention, whereas nutritional support was the most frequently identified additional need. Collaboration with nutrition professionals was rare, and fewer than half reported team-based treatment. The main barriers were lack of knowledge, lack of workplace training, and limited exposure to internal sarcopenia-related education. **Conclusions**: Despite high awareness, sarcopenia-related practice among Korean physical therapists remained limited. These findings highlight the need for standardized screening and treatment guidelines, structured continuing education programs, workplace-based training opportunities, and reimbursement policies that support routine sarcopenia screening and management.

## 1. Introduction

Modern society is rapidly aging, driven by a combination of increasing life expectancy and declining birth rates. This presents new challenges in managing the health of older adults [1,2]. Among these, sarcopenia is recognized as a disease or syndrome characterized by loss of muscle mass and reduced muscle strength and physical function, rather than a normal part of aging [3,4].

Sarcopenia is directly related not only to an increased risk of falls in older adults, decreased ability to perform activities of daily living, decreased quality of life, and increased hospitalization rates, but also ultimately to increased healthcare costs and mortality. Therefore, sarcopenia is a major task at the public health level that requires active screening, prevention, and effective intervention [5,6]. Major treatment and prevention strategies for sarcopenia are known to be nutritional interventions to promote muscle protein synthesis and exercise interventions for functional recovery and muscle strength enhancement [7].

In Korea, the aging population has grown rapidly; according to Statistics Korea, individuals aged 65 years and older accounted for approximately 18.4% of the total population in 2023, and this proportion is projected to exceed 40% by 2050 [8]. Against this backdrop, the prevalence of sarcopenia among Korean older adults has been reported to range from approximately 6.8% to 19.9%, depending on the diagnostic criteria and study population applied [9,10]. Notably, a nationwide survey based on the 2022 Korea National Health and Nutrition Examination Survey (KNHANES) found that the prevalence increased markedly with age, reaching 21.5% in men and 25.9% in women aged 80 years and older [10]. Furthermore, South Korea officially recognized sarcopenia as a distinct disease entity (ICD-10 code M62.5) in 2021, underscoring its growing clinical and policy relevance [11].

In particular, physical therapists are emphasized for the importance of their role as key experts who design and apply individualized resistance exercise and functional training programs for high-risk groups and patients with sarcopenia. The effective performance of physical therapists’ role is an essential factor in determining the prognosis of patients with sarcopenia [12]. However, physical therapists’ clinical practice in sarcopenia management is influenced by the complex interactions among their level of knowledge, their practice behaviors applied in actual clinical settings, and barrier factors that hinder the performance of practice [13]. This interplay among knowledge, clinical behavior, and systemic barriers aligns with established clinical implementation frameworks, such as the Knowledge-to-Action (KTA) framework, which posits that bridging the gap between evidence and practice requires not only individual knowledge acquisition but also organizational and policy-level enablers [14].

Previous studies have reported that the level of knowledge of healthcare professionals directly affects the quality of diagnosis and treatment, and that, when clinical barriers exist, even a high level of knowledge can limit the application of this knowledge in actual practice. This phenomenon, commonly referred to as the knowledge-to-practice gap, has been widely documented across various healthcare disciplines and represents a central challenge in evidence-based practice implementation [15,16]. In the survey study of specialists in China by Lu et al. [17] and the study conducted among specialists in the United States by Guralnik et al. [18], it was reported that physicians in internal medicine, orthopedics, and family medicine showed insufficient levels of sarcopenia-related knowledge compared with specialists in geriatrics, physical therapy, and rehabilitation medicine, resulting in limitations in clinical application. Verstraeten et al. [13] reported that, as a result of conducting a study on the perceptions, practices, barriers, and support of Dutch healthcare professionals regarding the clinical implementation of sarcopenia diagnosis and treatment, although the level of knowledge was high, screening and diagnosis were almost absent in clinical practice.

Most studies published to date have primarily focused on medical specialists or broad groups of healthcare professionals [13,17,18]. Notably, there is a significant lack of research that comprehensively investigates three key factors—knowledge, clinical practice, and barriers—and analyzes their interrelationships while reflecting the characteristics of the Korean healthcare environment and the physical therapy profession. In Korea specifically, although physical therapists serve as frontline practitioners in musculoskeletal and geriatric rehabilitation, no study has systematically examined how these three factors interact to shape sarcopenia-related practice among this professional group. Therefore, this study aimed to identify knowledge, clinical practice, and barriers related to sarcopenia among Korean physical therapists and to establish practical evidence for future improvements in education and policies. We hypothesized that, despite adequate awareness of sarcopenia, Korean physical therapists would demonstrate a significant gap between knowledge and clinical practice, and that specific individual and institutional barrier factors would be associated with reduced implementation of sarcopenia screening and treatment in clinical settings.

## 2. Materials and Methods

### 2.1. Study Design

This study was a descriptive cross-sectional survey conducted between 14 August 2025 and 31 December 2025, in the Republic of Korea. The study was conducted in accordance with the Declaration of Helsinki and was approved by the Public Institutional Bioethics Committee, Republic of Korea Approval No. P01-202503-01-008.

### 2.2. Participants

The sample size for this study was calculated using OpenEpi version 3.01 (Emory University, Atlanta, GA, USA; https://www.openepi.com, accessed on 14 August 2025), based on the standard formula for estimating a population proportion with finite population correction. The target population was defined as 50,800 physical therapists working in clinical settings in Korea as of the second quarter of 2024. A 95% confidence level, a 5% margin of error, an anticipated proportion of 50%, and a design effect of 1.0 were applied. The minimum required sample size was 382 participants. To allow for potential dropout, nonresponse, and incomplete responses in the online survey, 10% was added, yielding a final target sample size of 420 participants.

As this study aimed to investigate sarcopenia-related knowledge, clinical practice, and perceived barriers among the broader clinical physical therapy workforce in Korea, participants were not restricted to a specific specialty area. The inclusion criteria for survey participants were physical therapists who were currently working in clinics, primary to tertiary hospitals, and related centers and had at least one year of clinical experience, or physical therapists who were not currently employed but were within one year of leaving their job. The exclusion criterion was physical therapists who worked only in educational or research facilities and had no clinical experience.

### 2.3. Survey Instrument Development and Content Validation

To administer a sarcopenia-related survey among Korean physical therapists, a survey instrument was developed through a two-step process. In Phase 1, previous studies that conducted sarcopenia-related surveys were reviewed to collect items and to construct a preliminary questionnaire. Subsequently, a qualitative survey was conducted with eight physical therapists to gather feedback on item clarity, the appropriateness of the questionnaire structure, and the need for additional items. The participants were purposively selected to ensure sufficient clinical experience for questionnaire development, and their clinical experience ranged from eight to twenty-seven years. The first version of the questionnaire was then developed based on their feedback [19].

In Phase 2, cognitive interviews were conducted to confirm whether the Phase 1 questionnaire was applicable to the Korean clinical setting and to explore additional factors. The cognitive interviews were conducted with eight physical therapists, and after identifying perceptions and barrier factors related to sarcopenia as well as areas for item improvement, the questionnaire was revised and the final version was finalized by reflecting the results [20]. The final questionnaire consisted of 8 demographic items covering sex, age, employment status, education level, clinical experience, workplace, position, and practice area, 6 items on knowledge of sarcopenia, 10 items on sarcopenia screening, 6 items on sarcopenia treatment, and 4 items on environmental factors related to sarcopenia.

### 2.4. Data Collection

This study was conducted primarily through an online survey of physical therapists in Korea. The survey link was distributed through online communities commonly used by physical therapists in Korea, including Naver Cafe, a popular Korean online community platform, and KakaoTalk open chat rooms. To minimize coverage bias related to online-only participation, a paper-based questionnaire with identical items was also provided when online participation was difficult. Participation was voluntary, and only those who agreed to participate in the study were able to respond. The survey took approximately 15 to 20 min to complete, and all responses were submitted immediately upon completion.

To reduce duplicate response bias, participants were asked to provide their mobile phone number and physical therapist license number, and responses with matching identifiers were excluded from the analysis. To reduce information bias due to careless responding, responses judged to be careless, such as selecting the same option for all items, were also excluded from the analysis. Mobile phone numbers and physical therapist license numbers were collected only to verify duplicate participation, were separated from the response data, de-identified, and processed so that no personal identifying information was included in the analysis. Participants could withdraw from the survey at any time during completion.

### 2.5. Data Analysis

The collected data were analyzed using IBM SPSS Statistics version 22.0 (IBM Corp., Armonk, NY, USA). The main variables of interest were sarcopenia-related knowledge, screening practices, treatment practices, and environmental factors. Participant characteristics included sex, age, employment status, education level, clinical experience, workplace, position, and practice area. All variables were analyzed using descriptive statistics and presented in tables or figures. Categorical variables were presented as frequencies and percentages, and continuous variables were presented as medians and interquartile ranges.

## 3. Results

### 3.1. Participant Characteristics

While the initial target sample size was 420, a total of 236 physical therapists completed the survey within the recruitment period. As there were no incomplete responses or dropouts, all 236 participants were included in the final analysis. The median age of the participants was 31 years [IQR 28–37.8]. The sex distribution was 102 male (43.2%) and 134 female (56.8%). The highest level of education was most commonly a bachelor’s degree, 136 (57.6%), followed by an associate degree, 52 (22.0%), a master’s degree, 40 (17.0%), and a doctoral degree, 8 (3.4%). The median clinical experience was 8 years [IQR 4.1–13.3]. Regarding workplace setting, general hospitals accounted for the largest proportion, 108 (45.7%), followed by clinics, 55 (23.3%), social welfare centers, 29 (12.3%), long-term care hospitals, 28 (11.9%), university hospitals, 8 (3.4%), other, 7 (3.0%), and public health centers, 1 (0.4%). Other settings included adult day care center, exercise rehabilitation center, nursing home, rehabilitation hospital, and Korean medicine hospital. The most common employment/position status was permanent employee, 170 (72.0%), followed by permanent staff in senior management, 28 (11.9%), permanent staff in middle management, 26 (11.0%), and fixed-term employee, 12 (5.1%). Practice areas were neurologic, 103 (43.6%), musculoskeletal, 92 (39.0%), geriatrics, 29 (12.3%), pediatrics, 9 (3.8%), and other, 3 (1.3%). Other practice areas included health administration, cancer rehabilitation, and aquatic therapy (Table 1).

### 3.2. Knowledge of Sarcopenia

**Figure 1:**
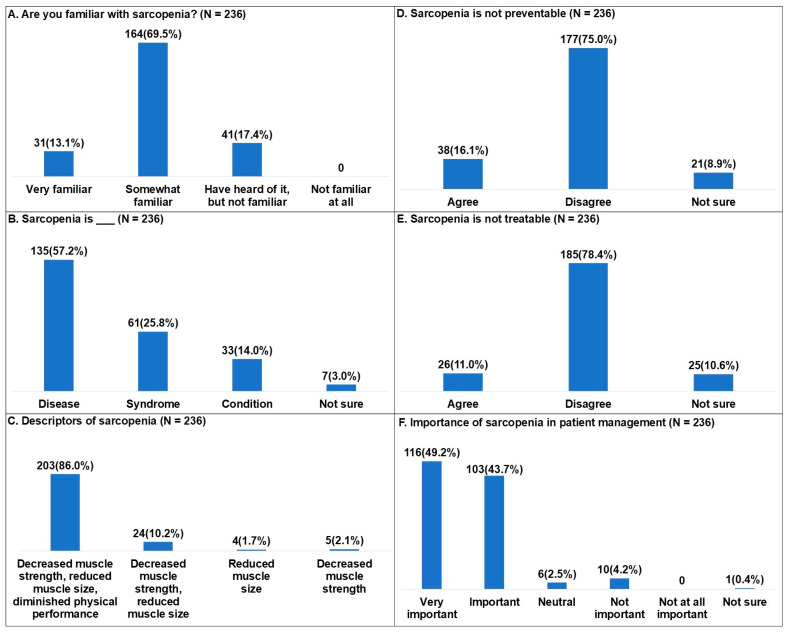
Knowledge of sarcopenia among Korean physical therapists.

In responses regarding awareness of sarcopenia, 164 participants reported that they were somewhat familiar (69.5%), 41 reported that they had heard of it but were not familiar (17.4%), and 31 reported that they were very familiar (13.1%) (Figure 1A). Regarding how sarcopenia was perceived, 135 participants identified it as a disease (57.2%), 61 as a syndrome (25.8%), 33 as a condition (14.0%), and 7 reported that they were not sure (3.0%) (Figure 1B).

For the question on descriptors of sarcopenia, 203 participants responded that it involves decreased muscle strength, reduced muscle size, and diminished physical performance (86.0%), 24 responded that it involves decreased muscle strength and reduced muscle size (10.2%), 5 responded that it involves decreased muscle strength (2.1%), and 4 responded that it involves reduced muscle size (1.7%) (Figure 1C).

Regarding preventability, for the question asking whether sarcopenia is not preventable, 177 participants disagreed (75.0%), 38 agreed (16.1%), and 21 reported that they were not sure (8.9%) (Figure 1D). In addition, regarding treatability, for the question asking whether sarcopenia is not treatable, 185 participants disagreed (78.4%), 26 agreed (11.0%), and 25 reported that they were not sure (10.6%) (Figure 1E).

Regarding the importance of sarcopenia in patient management, 116 participants responded that it was very important (49.2%), 103 responded that it was important (43.7%), 10 responded that it was not important (4.2%), 6 responded that it was neutral (2.5%), and 1 reported that they were not sure (0.4%) (Figure 1F).

### 3.3. Screening of Sarcopenia

**Figure 2:**
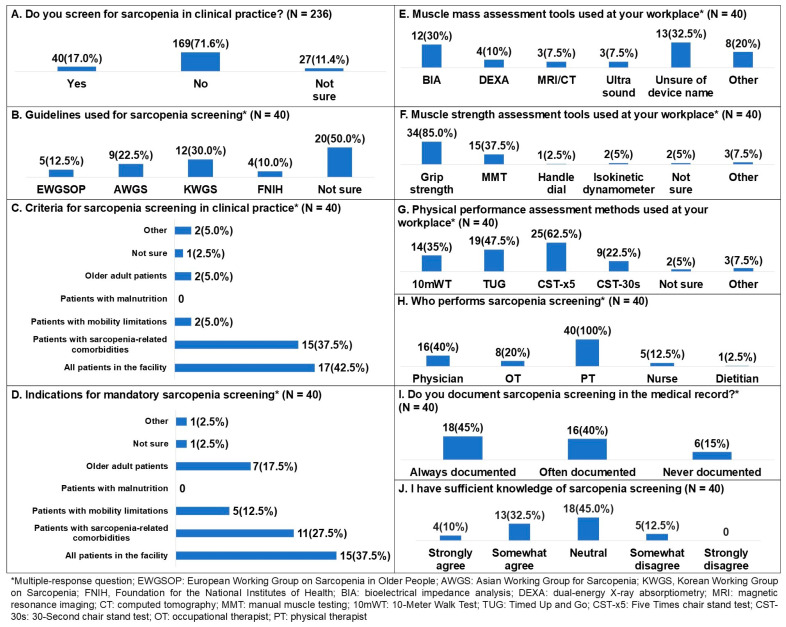
Screening for sarcopenia among Korean physical therapists.

For the question, “Do you screen for sarcopenia in clinical practice?”, responses were no 169 (71.6%), yes 40 (17.0%), and not sure 27 (11.4%) (Figure 2A). Additional questions were administered to physical therapists who reported screening for sarcopenia. Regarding the guidelines used for sarcopenia screening, 20 participants were unsure which guideline was being followed (50.0%), 12 indicated that the Korean Working Group on Sarcopenia (KWGS) guideline was being followed (30.0%), 9 indicated that the Asian Working Group for Sarcopenia (AWGS) guideline was being followed (22.5%), 5 indicated that the European Working Group on Sarcopenia in Older People (EWGSOP) guideline was being followed (12.5%), and 4 indicated that the Foundation for the National Institutes of Health (FNIH) guideline was being followed (10.0%) (Figure 2B).

Perceptions regarding the criteria for patients who receive sarcopenia screening in clinical practice were all patients in the facility 17 (42.5%), patients with sarcopenia-related comorbidities 15 (37.5%), patients with mobility limitations 2 (5.0%), older adult patients 2 (5.0%), other 2 (5.0%), not sure 1 (2.5%) (Figure 2C). Other target criteria included all patients and patients on absolute bed rest for 2 weeks or longer. For the question on which groups should be considered important targets for mandatory sarcopenia screening, responses were all patients in the facility 15 (37.5%), patients with sarcopenia-related comorbidities 11 (27.5%), older adult patients 7 (17.5%), patients with mobility limitations 5 (12.5%), not sure 1 (2.5%), other 1 (2.5%) (Figure 2D). Other target criteria included all patients.

The tools used in the workplace to assess muscle mass were unsure of device name 13 (32.5%), bioelectrical impedance analysis (BIA) 12 (30.0%), other 8 (20.0%), dual-energy X-ray absorptiometry (DEXA) 4 (10.0%), magnetic resonance imaging (MRI) or computed tomography (CT) 3 (7.5%), and ultrasound 3 (7.5%) (Figure 2E). Other responses included InBody, no device use, not assessing muscle mass, questionnaires, and functional assessment. The tools used to assess muscle strength were grip strength 34 (85.0%), manual muscle testing (MMT) 15 (37.5%), other 3 (7.5%), isokinetic dynamometer 2 (5.0%), not sure 2 (5.0%), and Handle dial 1 (2.5%) (Figure 2F). Other responses included InBody and Sarco. Methods used to assess physical performance were five times chair stand test (CST-x5) 25 (62.5%), timed up and go (TUG) 19 (47.5%), 10-Meter Walk Test (10 mWT) 14 (35.0%), 30 s chair stand test (CST-30s) 9 (22.5%), other 3 (7.5%), and not sure 2 (5.0%) (Figure 2G). Other methods included 4 m Walk Test (4 mWT), short physical performance battery (SPPB), and one leg stance test. Perceptions regarding which profession is responsible for sarcopenia screening were physical therapist (PT) 40 (100.0%), physician 16 (40.0%), occupational therapist (OT) 8 (20.0%), nurse 5 (12.5%), and dietitian 1 (2.5%) (Figure 2H).

For the question on whether sarcopenia screening results were documented in the medical record, responses were always documented 18 (45.0%), often documented 16 (40.0%), and never documented 6 (15.0%) (Figure 2I). For the question on having knowledge about sarcopenia screening, responses were neutral 18 (45.0%), somewhat agree 13 (32.5%), somewhat disagree 5 (12.5%), strongly agree 4 (10.0%) (Figure 2J).

### 3.4. Treatment of Sarcopenia

**Figure 3:**
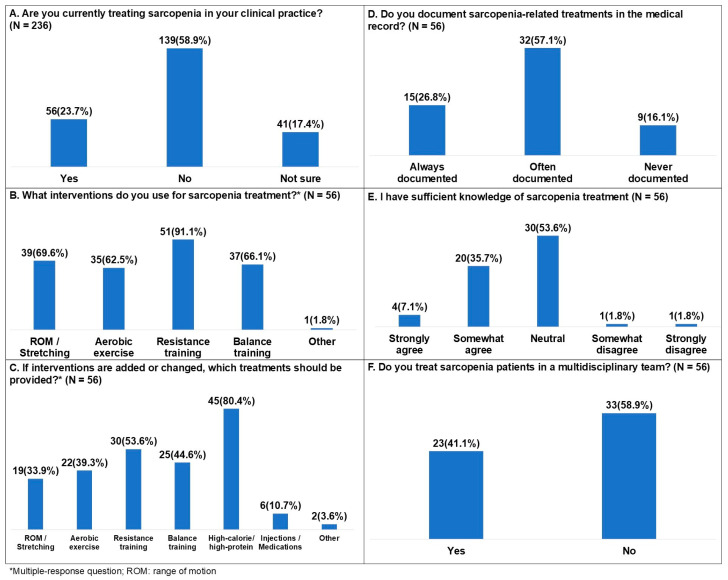
Treatment of sarcopenia among Korean physical therapists.

For the question on whether participants were currently providing treatment for sarcopenia, responses were no 139 (58.9%), yes 56 (23.7%), and not sure 41 (17.4%) (Figure 3A). Additional questions were administered to physical therapists who reported treating sarcopenia.

Among physical therapists who reported treating sarcopenia, the intervention approaches currently applied were resistance training 51 (91.1%), range of motion (ROM) exercises and stretching 39 (69.6%), balance training 37 (66.1%), aerobic exercise 35 (62.5%), and other 1 (1.8%) (Figure 3B). Other interventions included robotic therapy. Regarding which treatments were needed if therapeutic interventions were added or changed, responses were high-calorie/high-protein 45 (80.4%), resistance training 30 (53.6%), balance training 25 (44.6%), aerobic exercise 22 (39.3%), ROM exercises and stretching 19 (33.9%), injections or medications 6 (10.7%), and other 2 (3.6%). Other treatments included psychological treatment and postural treatment (Figure 3C).

For the question on whether sarcopenia-related treatments were documented in the medical record, responses were often documented 32 (57.1%), always documented 15 (26.8%), and never documented 9 (16.1%) (Figure 3D). For the question on sufficient knowledge of sarcopenia treatment, responses were neutral 30 (53.6%), somewhat agree 20 (35.7%), strongly agree 4 (7.1%), somewhat disagree 1 (1.8%), and strongly disagree 1 (1.8%) (Figure 3E). For the question on whether participants treated patients with sarcopenia in a multidisciplinary team, responses were no 33 (58.9%) and yes 23 (41.1%) (Figure 3F).

### 3.5. Sarcopenia Care Environment

**Figure 4:**
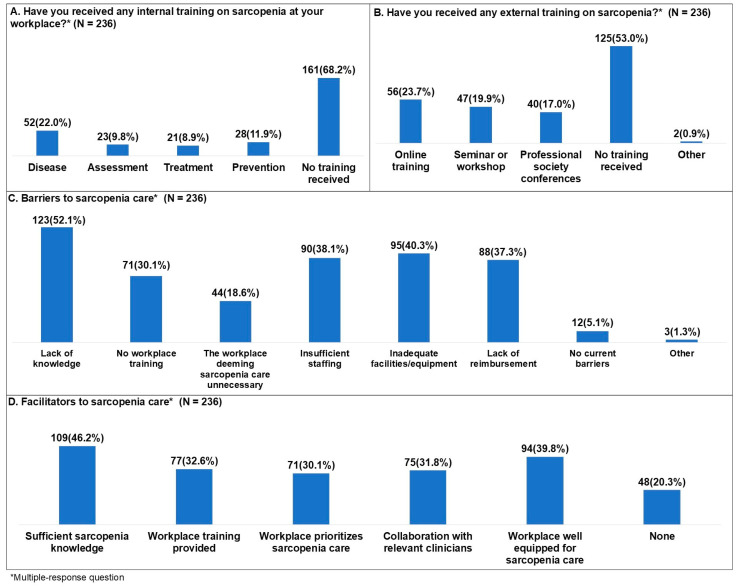
Care environment of sarcopenia among Korean physical therapists.

For the question on whether participants had received internal training related to sarcopenia at their workplace, responses were no training received 161 (68.2%), education about the disease 52 (22.0%), education about prevention 28 (11.9%), education about assessment 23 (9.8%), and education about treatment 21 (8.9%) (Figure 4A). For external training related to sarcopenia received outside the workplace, responses were no training received 125 (53.0%), online training 56 (23.7%), seminars or workshops 47 (19.9%), professional society conferences 40 (17.0%), and other 2 (0.9%) (Figure 4B). Other responses included school and continuing education.

Barriers to sarcopenia care were lack of knowledge 123 (52.1%), inadequate facilities/equipment 95 (40.3%), insufficient staffing 90 (38.1%), lack of reimbursement 88 (37.3%), no workplace training 71 (30.1%), the workplace deeming sarcopenia care unnecessary 44 (18.6%), no current barriers 12 (5.1%), and other 3 (1.3%) (Figure 4C). The other responses included inability to reassess, unclear management systems for exercise and nutrition, and difficulty performing accurate assessment.

Facilitators to sarcopenia care were sufficient sarcopenia knowledge 109 (46.2%), workplace well equipped for sarcopenia care 94 (39.8%), workplace training provided 77 (32.6%), collaboration with relevant clinicians 75 (31.8%), workplace prioritizes sarcopenia care 71 (30.1%), and none 48 (20.3%) (Figure 4D).

## 4. Discussion

This study was conducted to identify the level of awareness of sarcopenia, the current status of clinical practice, and barriers among Korean physical therapists. In the survey on awareness of sarcopenia, most participants, 82.6%, reported being aware of sarcopenia, and 92.8% responded that sarcopenia was a very important or important disease. Together with positive perceptions that sarcopenia is preventable and treatable, 75.0% and 78.4%, respectively, these findings suggest that physical therapists view sarcopenia as an area requiring active intervention. In addition, 86.0% of participants correctly understood the definition of sarcopenia by including decreased muscle strength, decreased muscle mass, and decreased physical performance, indicating that the basic level of knowledge was sufficient.

In the survey on sarcopenia screening and treatment, in contrast to the high level of awareness, only 17.0% of participants reported performing sarcopenia screening in clinical practice, and the proportion directly providing treatment also remained at 23.7%. This awareness–practice gap can be examined from three structural perspectives: educational, clinical–organizational, and health policy dimensions. From an educational standpoint, the findings reflect an insufficient translation of declarative knowledge into procedural competence—a pattern consistent with the knowledge-to-practice gap described in the literature [15]. From a clinical–organizational perspective, the absence of institutionally embedded sarcopenia screening protocols suggests that organizational structures have not yet adapted to support evidence-based implementation. From a health policy standpoint, the lack of reimbursement mechanisms for sarcopenia screening and individualized exercise interventions creates systemic disincentives that further impede clinical uptake. When considered alongside international evidence—including a study of healthcare professionals in Singapore reporting that high awareness does not translate into screening in practice, studies in Australia and New Zealand reporting low rates of diagnosis and treatment due to a lack of diagnostic tools, and reports of limited management capacity among medical specialists in Türkiye due to insufficient awareness—this pattern suggests that not only in Korea but also in many other countries, sarcopenia is well defined academically but remains in a transitional stage before being fully established as a standardized clinical workflow [4,21,22]. A recent multicenter study in China similarly demonstrated that, despite positive attitudes toward sarcopenia, practical engagement remained low, attributing this to regional disparities in healthcare resources and infrastructure [23]. In particular, even among those who reported performing screening, 50% conducted screening without clearly recognizing internationally accepted guidelines—a notable issue that indicates current sarcopenia management is being carried out in a fragmented manner without standardized protocols.

An interesting finding of this survey is that physical therapists perceived their role in sarcopenia screening in a proactive manner. In the question regarding which profession is responsible for sarcopenia screening, physical therapists were identified at the highest rate, 100%, which was substantially higher than physicians, 40%, and occupational therapists, 20%. This suggests that there is an internal consensus that physical therapists, as exercise experts, are well suited to lead the entire process from sarcopenia screening to resistance exercise and functional training. Considering the need to design and deliver exercise programs through various approaches, strategies to promote sustained participation, reinforcement of positive messages about exercise, and provision of education on exercise and its related benefits, physical therapists can be considered the most appropriate professionals for sarcopenia treatment [24]. This professional identity is consistent with evidence that exercise specialists who receive targeted training demonstrate higher rates of sarcopenia screening and intervention [12].

Regarding the current treatment status, physical therapists not only reported implementing resistance exercise (91.1%) but also recognized a high need for nutritional interventions such as the provision of high-calorie and high-protein foods (80.4%). However, although physical therapists recognized a great need for nutritional interventions, recognition of dietitians as professionals involved in sarcopenia screening was very low (2.5%), and the proportion of team-based treatment also remained below half (41.1%). Compared with the emphasis on the need for a multidisciplinary approach reported in studies of healthcare professionals in Australia and New Zealand, the Korean clinical environment can be considered to still show a strong tendency for each professional group to operate independently [25]. Similar cases have also been reported internationally. According to a study examining attitudes toward collaborative care between dietitians and physical therapists, both professions viewed collaborative treatment positively and were willing to engage in it. However, barriers to collaboration were reported to include insufficient communication between professionals, difficulty securing additional treatment resources, and issues related to financial reimbursement [26].

In addition, other studies have reported that, although the importance of integrated patient communication for addressing sarcopenia is well recognized, implementation remains limited due to restricted access to nutrition services in primary care settings and a lack of knowledge and awareness [27,28]. To address this issue, it is important to understand the perspectives of different professionals. The barriers identified in this study can be understood as operating across three interconnected levels: at the individual level, insufficient knowledge and confidence; at the organizational level, lack of standardized protocols, inadequate staffing, and insufficient facilities; and at the systemic level, absence of reimbursement and limited interprofessional collaboration mechanisms. Addressing these multilevel barriers requires coordinated responses at each level rather than isolated interventions. For multidisciplinary management of sarcopenia, it is necessary to establish regular interprofessional exchange and a clear communication system. Factors that may be considered include holding regular in-person communication and meetings, ensuring close proximity among professionals, providing financial reimbursement for professionals, and involving family members and caregivers in meetings [28].

In the survey on the practice environment and barriers, specific barriers were identified that prevented physical therapists from translating their adequate awareness of the importance of sarcopenia management into clinical practice. The most frequently reported limiting factor was lack of knowledge (52.1%), which is closely related to the response that the workplace does not provide relevant training (30.1%). In fact, 68.2% of respondents reported having no experience of receiving sarcopenia-related education within their workplace. This suggests that, although individual interest among physical therapists is high, systematic job-related training at the institutional level is insufficient. In particular, the finding that lack of knowledge was identified as the largest barrier supports the urgency of specialized continuing education to bridge the gap between awareness and actual implementation in clinical practice. A lack of knowledge for clinical use is not merely an individual issue but represents a type of knowledge-to-practice gap that exists widely across global health systems. It has also been reported that, on average, it takes 17 years for evidence-based practice to be implemented in clinical settings [15].

To bridge this gap, an active introduction of continuing medical education systems is needed. A meta-analysis on continuing medical education reported effects of 79% on knowledge acquisition and 58% on changes in clinical behavior [29]. In particular, improved knowledge was shown to increase confidence, leading to actual changes in clinical behavior [30]. Considering findings that relevant knowledge improved immediately after medical education but declined again after six months, one-time education alone is insufficient to resolve the knowledge-to-practice gap. Therefore, it is necessary to establish a specialized continuing education system on sarcopenia to strengthen knowledge continuously [4]. Another key barrier identified by physical therapists was the absence of reimbursement (37.3%). Within the current Korean healthcare system, the lack of clear reimbursement items for sarcopenia screening or individualized resistance exercise interventions may lead hospital management to regard sarcopenia management as unnecessary (18.6%). This in turn creates a vicious cycle that leads to insufficient staffing (38.1%) and inadequate facilities or environment (40.3%). A large-scale analysis of healthcare institutions in the United States confirmed that increases in performance incentives linearly increased program participation, indicating that reimbursement structures directly influence healthcare professional behavior [31]. Moreover, an increase in the use of medications following the expansion of National Health Insurance coverage for osteoporosis treatments in Korea suggests that the same mechanism operates within the Korean healthcare system [32]. Therefore, without institutional reimbursement support, it can be understood that clinical implementation is likely to be critically hindered in real-world practice.

This study is meaningful as an early study that assessed overall perceptions of sarcopenia among Korean physical therapists. However, it has several limitations. First, the estimated required sample size was 420, but only 236 participants were included in the final analysis, and the target was not reached. Therefore, caution is needed when generalizing the findings to the entire population of physical therapists in Korea. Second, because recruitment was conducted primarily through voluntary participation in online communities, self-selection bias cannot be excluded. In addition, uneven participation across geographic settings, such as urban versus rural areas, may have influenced the findings and limited the generalizability of the results. Third, given the nature of surveys relying on respondents’ subjective judgments, actual knowledge levels or clinical practice competence may have been overestimated or underestimated. Fourth, because the questionnaire was designed to capture descriptive information across multiple distinct domains rather than a single latent construct, one overall internal consistency estimate was not calculated, and formal reliability testing was not performed. Therefore, the findings should be interpreted with caution.

## 5. Conclusions

This study investigated the perceptions and current practice status of Korean physical therapists, who are key personnel in sarcopenia management in a rapidly aging society. The findings showed that Korean physical therapists recognized sarcopenia as an important disease and had a strong professional identity as treatment experts. However, clinical implementation remains low due to insufficient knowledge and institutional limitations. These findings suggest that the dissemination of standardized guidelines, the development of systematic continuing education programs, and the establishment of a realistic reimbursement framework may help bridge the gap between awareness and practice. The results of this study may serve as foundational data for future geriatric health policy development and for informing strategies to strengthen the professional role of physical therapy in sarcopenia management.

## Figures and Tables

**Table 1:** Demographic and professional characteristics of the survey respondents (N = 236).

Characteristics		N (%) or Median [Interquartile Range]
Age (y)		31 [28–37.8]
Sex	Male	102 (43.2%)
	Female	134 (56.8%)
Highest education level	Associate degree	52 (22.0%)
	Bachelor’s degree	136 (57.6%)
	Master’s degree	40 (17.0%)
	Doctoral degree	8 (3.4%)
Clinical experience (y)		8 [4.1–13.3]
Employment setting	Clinic	55 (23.3%)
	General hospital	108 (45.7%)
	University hospital	8 (3.4%)
	Long-term care hospital	28 (11.9%)
	Public health center	1 (0.4%)
	Social welfare center	29 (12.3%)
	Other	7 (3.0%)
Employment/position status	Fixed-term employee	12 (5.1%)
	Permanent employee	170 (72.0%)
	Permanent staff in middle management	26 (11.0%)
	Permanent staff in senior management	28 (11.9%)
Practice area	Musculoskeletal	92 (39.0%)
	Neurologic	103 (43.6%)
	Pediatrics	9 (3.8%)
	Geriatrics	29 (12.3%)
	Other	3 (1.3%)

y: years.

## Data Availability

The dataset used and analyzed in this study is available from the corresponding author. The data are not publicly available due to privacy concerns and ethical restrictions, as the study involved the collection of personal identifiers from participants and was conducted under institutional bioethics committee approval.

## Abbreviations

The following abbreviations are used in this manuscript:

EWGSOP	European Working Group on Sarcopenia in Older People
AWGS	Asian Working Group for Sarcopenia
KWGS	Korean Working Group on Sarcopenia
FNIH	Foundation for the National Institutes of Health
BIA	Bioelectrical impedance analysis
DEXA	Dual-energy X-ray absorptiometry
MRI	Magnetic resonance imaging
CT	Computed tomography
MMT	Manual muscle testing
10 mWT	10-Meter Walk Test
TUG	Timed Up and Go
CST-x5	Five Times chair stand test
CST-30	30-Second chair stand test
OT	Occupational therapist
PT	Physical therapist
ROM	Range of motion
